# Dynamic millimeter-wave OAM beam generation through programmable metasurface

**DOI:** 10.1515/nanoph-2021-0790

**Published:** 2022-02-17

**Authors:** Xudong Bai, Fuli Zhang, Li Sun, Anjie Cao, Chong He, Jin Zhang, Weiren Zhu

**Affiliations:** School of Microelectronics, Northwestern Polytechnical University, Xi’an, 710129, China; Shanghai Aerospace Electronics Co., Ltd., Shanghai, 201821, China; School of Physical Science and Technology, Northwestern Polytechnical University, Xi’an, 710129 China; Shanghai Institute of Satellite Engineering, Shanghai, 201109, China; Department of Electronic Engineering, Shanghai Jiao Tong University, Shanghai, 200240, China

**Keywords:** metasurface, mmWave, orbital angular momentum, programmable, reflective

## Abstract

Millimeter-wave (mmWave) and orbital angular momentum (OAM) multiplexing are two key technologies for modern wireless communications, where significant efforts have been devoted to combining these two technologies for extremely high channel capacities. Recently, programmable metasurfaces have been extensively studied for stimulating dynamic multi-mode OAM beams, owing to their ability of subtle dynamic modulation over electromagnetic waves in a digital manner. However, programmable metasurfaces for mmWave OAM stimulation are rarely mentioned, due to the requirement of extremely high processing precision for mmWave applications. In this paper, a programmable metasurface is presented to stimulate dynamic multi-mode mmWave vortex beams. The proposed metasurface is composed of electronically reconfigurable units, which is obtained through configuration integration of a PIN diode within each radiation patch for modulating the unit resonant property. Both low reflection losses and stabilized inverse phase states are obtained for the binary unit coding states within the operation band. Through modulating the real-time coding distribution on the metasurface by programmable bias circuit, the generation of mmWave OAM beams with mode numbers *l* = 0, *l* = +1, *l* = +2, and *l* = +3 are numerically designed and experimentally verified. Our study paves a new perspective for the cross amalgamation of both mmWave and multi-mode OAM technologies.

## 1 Introduction

With the rapid development of smart terminals and internet of things, large-bandwidth, and high-speed transmissions are highly demanded in modern wireless communications [1]. In the fifth-generation communication, the wideband characteristics of millimeter wave (mmWave) band have already been thoroughly studied and put into deployments, due to the rich spectrum resources [2, 3]. The next-generation wireless systems are constructed through mmWave hybrid technology to dramatically increase the data rates and augment the overall system capacity [4]. Later, mmWave technologies associated with massive MIMO and cellular communications are also introduced to expand the transmission scale and enhance service quality [5, 6]. In all these applications, smart antenna arrays with the ability of dynamic directional beamforming or beam scanning are indispensable, bringing enormous expenses based on the conventional phased array systems [7, 8]. On the other hand, vortex beams associated with orbital angular momentum (OAM) technology have also become a key promoter for the next-generation wireless communications, owing to its multimode orthogonal characteristic and potential improvement in channel capacity and spectrum efficiency [9]. OAM is known as one kind of angular momentum, which is associated with the vortex electromagnetic (EM) wavefront and a rotational phase profile of exp(i*ℓθ*), where *ℓ* is the OAM topological charge and *θ* is the azimuth angle [10, 11]. Vortex beams with different OAM integral topological charges are orthogonal and independent with each other, and multiplex system for information transmission can thus be constructed over the same spectral channel, which brings a tremendous increase in spectrum efficiency and channel capacity [12, 13]. Owing to this unique orthogonal property, multiplex OAM has been widely studied and implemented in microwave domain [14, 15]. Later, broadband fixed-mode OAM beams are stimulated in mmWave domain through code metasurfaces [16, 17], but the OAM application for channel multiplexing has been strongly restricted, since the OAM mode cannot be modulated dynamically. Therefore, bright prospects could be apparently expected with a combination of both mmWave and dynamic multimode OAM multiplexing for better utilizing the EM spectrum [18, 19].

Many elegant design methods have been proposed for stimulating dynamic vortex beams, including reconfigurable antenna arrays [20], circular phased arrays [21], and programmable metasurfaces [22], [23], [24]. Particularly, programmable metasurfaces [25], [26], [27], [28] are artificially engineered reconfigurable planar structures with unprecedented capacity for the neat manipulation of EM waves. Compared with conventional phased array systems using digital or analog phase shifters, programmable metasurfaces show great advantages on reducing the complexity and cost for dynamic beam forming or scanning [29], [30], [31], [32], [33]. Recently, programmable metasurfaces have been thoroughly studied for stimulating dynamic vortex waves. 1 bit or 2 bit programmable reflective metasurfaces were constructed by integrating either PIN diodes or voltage-controlled varactor diodes to form the reconfigurable multi-mode OAM generators in C-band or X-band [34], [35], [36]. A programmable 3 bit reflective metasurface was introduced to manipulate the vortex phase front in both space and time and thus obtain the multifunctional vortex beams in X-band [37]. Later, dynamic C-band vortex beams with mode-reconfigurable and frequency-adjustable integration capabilities were produced through programmable reflective metasurface, each unit of which is composed of an octagonal ring slot and a varactor diode [38]. To avoid the feed blockage in reflective mode, a transmissive metasurface was introduced to stimulate dynamical multi-mode vortex beams and provide good spatial coverage for oblique EM incidences in X-band [39]. However, for all these designs, the programmable metasurfaces are operating at the low-frequency microwave domain, and programmable metasurfaces for stimulating mmWave OAM beams have not been presented to the best of our knowledge, due to the requirement of extremely high processing precision in mmWave applications, since metasurface in mmWave band is usually with a smaller dimension and thus more sensitive to the manufacturing process accuracy, when comparing with conventional microwave metasurfaces [40, 41].

In this paper, a reflective programmable metasurface is proposed to stimulate dynamic multi-mode mmWave vortex beams. The programmable metasurface consists of electronically adjustable units with two phase states (0/*π*), which are achieved by integrating a PIN diode in each radiation patch to modulate the unit resonant property. Through modulating the real-time coding distribution on the metasurface by a programmable bias circuit, dynamic multi-mode mmWave vortex beams can be generated. Moreover, in order to verify the proposed concept, a dynamic reflective programmable metasurface operating at 28–30 GHz with 20 × 20 units is designed and fabricated, which can generate four-mode adjustable vortex beams, including OAM modes *l* = 0, *l* = +1, *l* = +2, and *l* = +3. The generation of all these four OAM beams is verified by both simulations and experiments, which demonstrates the effectiveness of our design. The proposed mmWave OAM metasurface may become an eligible candidate for the hybrid communications of both mmWave and multi-mode OAM technologies in the future.

## 2 Theoretical design

**Figure 1:**
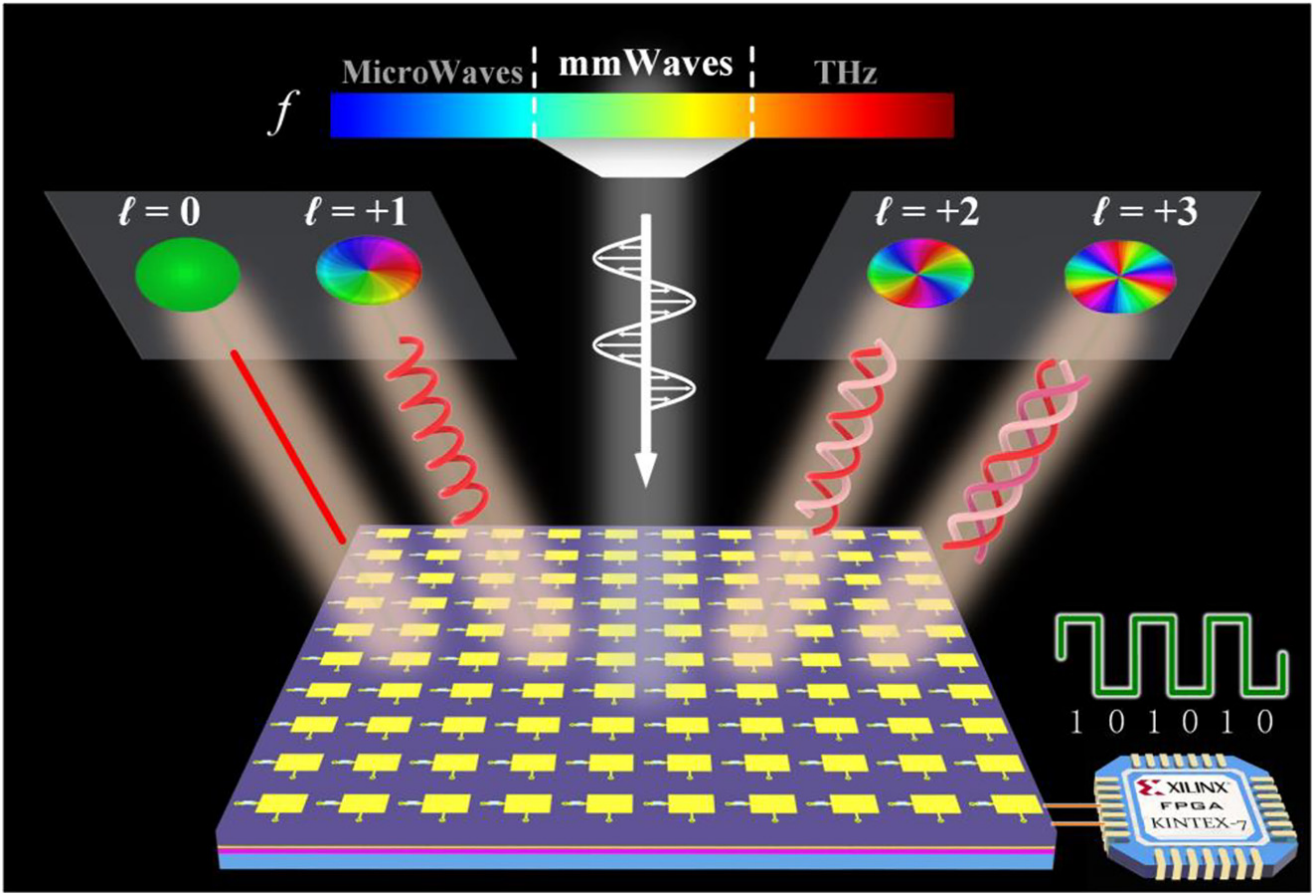
Schematic diagram of the programmable mmWave metasurface for dynamic OAM beam generation.

The overall schematic diagram of the proposed mmWave metasurface system for stimulating multiple OAM beams is shown in Figure 1. A standard horn antenna is employed as the feed source and positioned in the central axis of the reflective metasurface, which emits the EM forward-traveling wave onto the metasurface. Each active metasurface unit serves as a 1 bit phase shifter for the modulation of the incoming EM wave, and mmWave vortex phase-fronts can thus be transformed in the reflected waves. Four types of vortex beams, including OAM modes *l* = 0, *l* = +1, *l* = +2, or *l* = +3, can be real-time stimulated by varying the code distributions on the metasurface, which could be dynamically modulated by biasing the PIN diodes of the units independently via a steering-logic board.

## 3 Metasurface unit design

**Figure 2:**
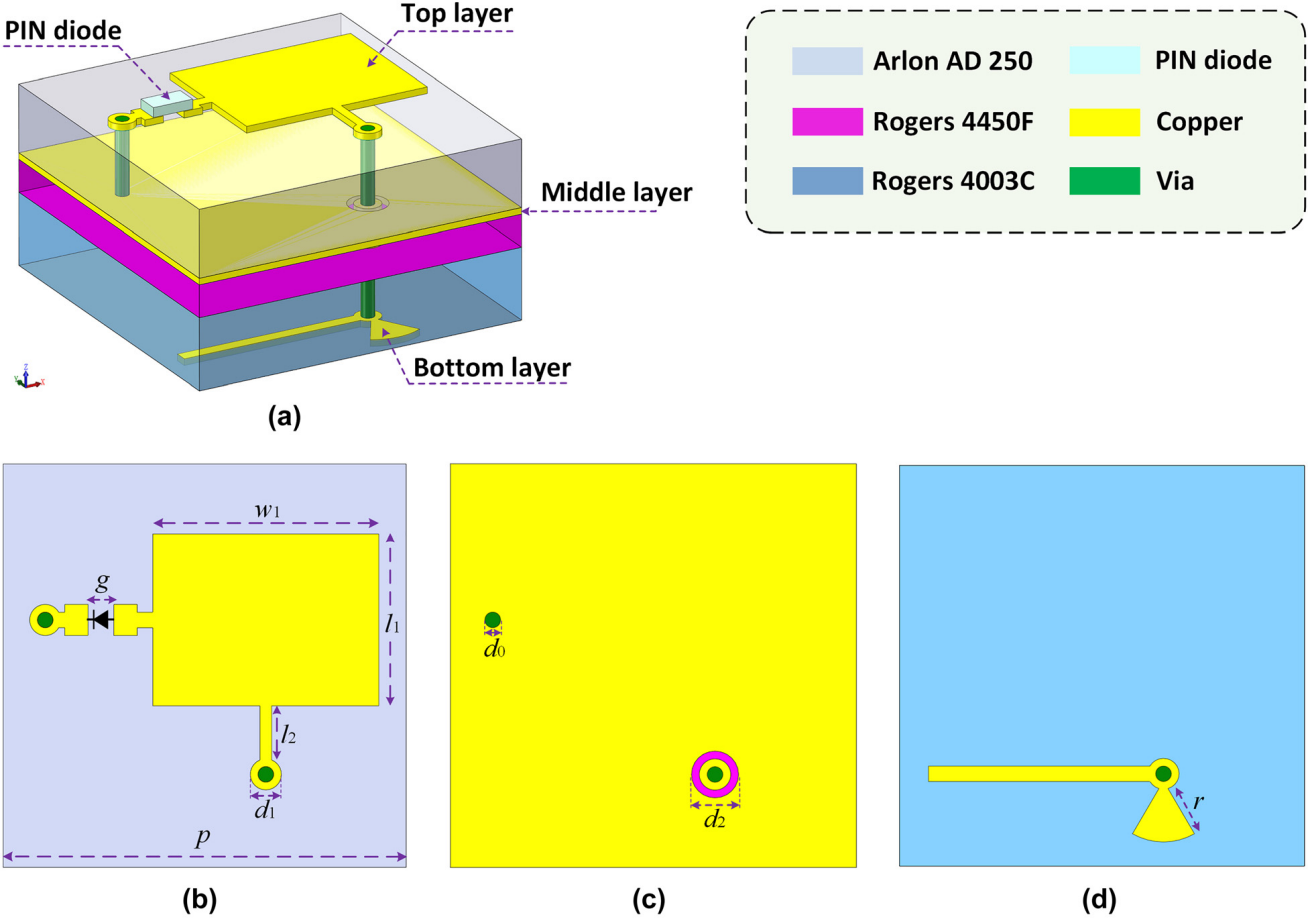
Topology of the reconfigurable metasurface unit. (a) 3D illustration. (b) Top radiating layer. (c) Middle ground layer. (d) Bottom bias layer. The unit is designed at the center frequency of 29 GHz with a periodicity of *λ*/2. Detailed parameters are as follows: *p* = 5.17, *g* = 0.33, *w*_1_ = 2.9, *l*_1_ = 2.2, *l*_2_ = 0.7, *d*_0_ = 0.2, *d*_1_ = 0.4, *d*_2_ = 0.5, and *r* = 0.68 (item: mm).

Figure 2 gives the topology of the presented programmable mmWave metasurface unit, which is further refined based on the classic architecture employing resonant tunable approach for 1 bit phase modulation, merely restricted in low-frequency microwave domain from S-band to Ku-band [27, 28, 36]. Since the mmWave metasurface is more sensitive to the manufacturing process accuracy, the concise design could be a good guarantee for the working performance even in case of an insufficient manufacturing accuracy. The unit consists of three copper layers and one PIN diode, which are supported by two substrate layers and one thin bonding film layer. The copper layers include, from top to bottom, a rectangular radiation patch, a metal ground plane, and a bias layer. The PIN diode is integrated at the end of the rectangular patch and connected with the ground plane by a shorting via in order to tune the patch’s resonance property through the bias layer, resulting in a phase difference of *π* in different modes for the reflected waves. In order to minimize the impact on the high frequency performance, the DC-bias line is set at the middle of the non-radiating patch edge. Besides, an open-ended radial stub is also integrated in the bias layer for isolating high frequency signals. The detailed geometrical parameters of the meta-unit are listed in the caption of Figure 2. The top substrate layer is Arlon AD250 (dielectric constant of 2.50, loss tangent of 0.0018, and thickness of 0.762 mm), the lower substrate layer is Rogers 4003C (dielectric constant of 3.55, loss tangent of 0.0027, and thickness of 0.508 mm), and the middle bonding film layer is Rogers 4450F (dielectric constant of 3.52, loss tangent of 0.004, and thickness of 0.203 mm).

The PIN diode, MACOM MADP-000907-14020 [42], is adopted to acquire lower unit insertion losses within the designed mmWave frequency band, which is modeled by the equivalent lumped components in series or parallel for the two states. For positive biasing, the proposed reconfigurable unit is operating at the *π*-state along with a typical series resistor *R*_*π*_ = 5.2 Ω and inductor *L*_*π*_ = 30 pH adopted for the PIN diode; while for negative biasing, the proposed reconfigurable unit is operating at the 0-state along with a typical parallel capacitance C_0_ = 0.025 pF and inductor L_0_ = 30 pH employed for the PIN diode.

**Figure 3:**
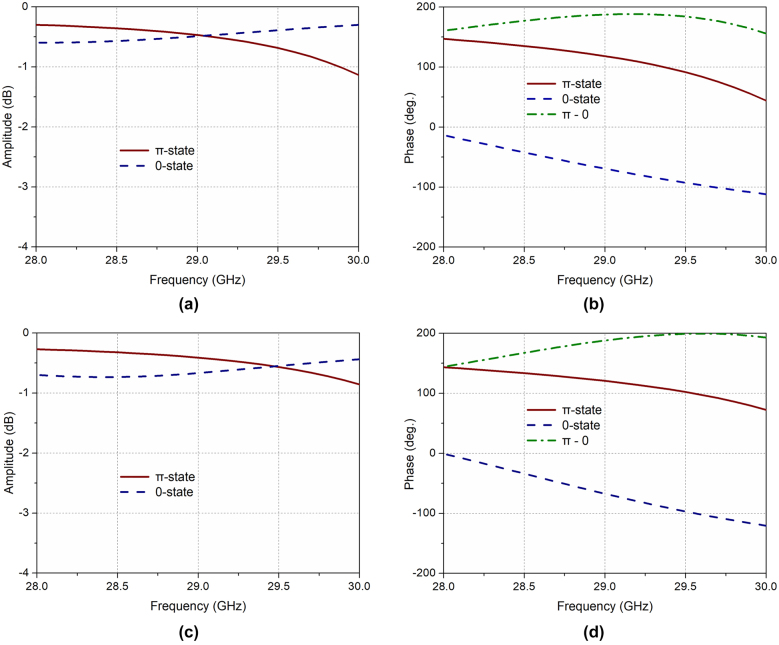
Simulated reflection coefficients of the unit. (a) Amplitude and (b) phase for both *π*/0 states with normal incidence. (c) Amplitude and (d) phase for both *π*/0 states with incidence angle *θ* = 30°.

The numerical simulation of the proposed mmWave unit is carried out with the help of the commercial software package CST Microwave Studio by using unit cell boundary conditions along with the Floquet-port excitations [43]. The simulated reflection coefficients of the metasurface unit, in terms of both amplitude and phase, for the binary states are given in Figure 3. For a normal incidence EM wave, the unit reflection loss is less than 1.1 dB (0.6 dB) from 28 to 30 GHz when the reconfigurable unit is operating at the *π*-state (0-state), as seen in Figure 3(a). Moreover, Figure 3(b) shows the perfect binary phase modulation with a stable phase difference around 180° for the two states in the entire band from 28 GHz to 30 GHz. The proposed reconfigurable unit also shows excellent angular tolerance. As seen in Figure 3(c) and (d), for an oblique incidence wave with incidence angle *θ* = 30°, the reflection losses are less than 0.8 dB while keeping stable phase difference between the binary states, with very small deviation within the operation frequency band. In general, the proposed mmWave metasurface unit can achieve excellent 1 bit phase modulation with very low reflection loss, making it appropriate for the overall programmable metasurface design.

## 4 OAM metasurface design

Based on the proposed reconfigurable unit, a programmable mmWave metasurface is designed and fabricated with 20 × 20 units, and the overall aperture is 103.4 mm × 103.4 mm. A standard waveguide horn (WR-28) is positioned in the central axis of the metasurface and utilized as the focal source for the metasurface aperture illumination, and the radiation gain of 11.8 dB along with the 3 dB beamwidth of 48° are obtained for the feed horn at 29 GHz. Since each unit of the metasurface is integrated with a PIN diode, a total of 400 PIN diodes are employed to provide the individual phase modulation through the bottom bias layer network.

**Figure 4:**
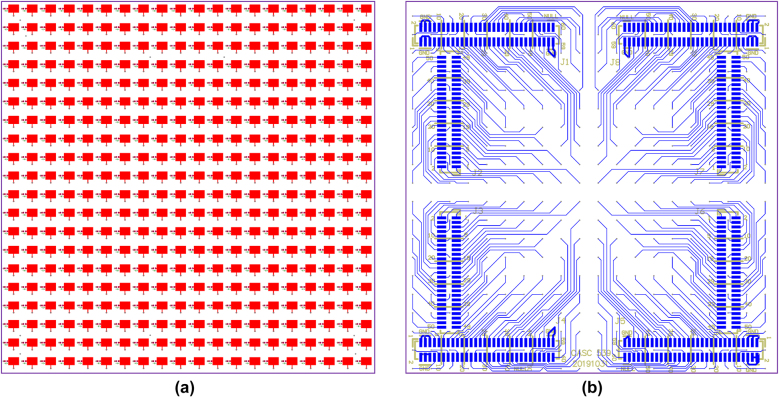
Overall configuration of the proposed metasurface with 20 × 20 units. (a) Top layer with the reflective patches. (b) Bottom layer with bias layer network layout.

The 3D topology diagram of the metasurface is shown in Figure 1. The top reflective metasurface patches along with the bottom bias layer network of the proposed metasurface are also provided in Figure 4. In order to simplify the bottom bias layer design layout and minimize the bias line interference, the bias layer network is divided into four symmetric parts, as shown in Figure 4(b).

As mentioned earlier, vortex beams have a rotational phase profile associated with azimuth angle, that is φ(θ)=φ(x,y)=l⋅arctan(y/x) , where (*x*, *y*) is the unit position coordinates on the metasurface. In order to stimulate the focused multi-mode vortex beams through the programmable metasurface, the optimal compensation phase distribution should be designed to satisfy the following equation [44]:

(1) $$\phi_{\text{OAM}}\left(x,y\right)=2\pi \left(\sqrt{\left(x^{2}+y^{2}\right)+F^{2}}-F\right)/\lambda +l\cdot \arctan\left(y/x\right),$$

where *l* is the designed OAM mode number, *λ* is the free-space wavelength at the designed frequency, and *F* is the focal length of the feed phase center. Moreover, since only binary phase states are available for the reconfigurable unit, the compensation phases should be furtherly quantized into two coding states according to

(2) $$\phi_{\text{QOAM}}=\left\{ \begin{array}{l} 0,\;\phi_{\text{OAM}}\in \left[0+2n\pi ,\pi +2n\pi \right)\\ \pi ,\;\phi_{\text{OAM}}\in \left[\pi +2n\pi ,2\pi +2n\pi \right) \end{array}\right.\;n\in Z\text{.}$$

In the conventional reflective metasurface designs, the feed location for the focal source should be elaborately designed, since the aperture efficiency is directly influenced by the focal length, which lies mainly on two factors, including the spillover efficiency and the illumination efficiency. The spillover efficiency attributes to the radiated power which is intercepted by the metasurface aperture relative to the total radiated power from the feeder, while the illumination efficiency is related to the asymmetrical distribution of the EM field caused by the field amplitude tapering across the metasurface [45]. When the feeder horn is set near the metasurface plane, an increscent spillover efficiency along with a decreased illumination efficiency would be produced. Correspondingly, the reduced spillover efficiency along with the increased illumination efficiency would be obtained for larger feed focal length. To make a tradeoff between the spillover efficiency and the illumination efficiency for a higher metasurface aperture efficiency, the feed horn antenna is placed with a focal length of *F* = 12*λ* = 62.04 mm, corresponding to an edge taper of approximate −10 dB.

**Figure 5:**
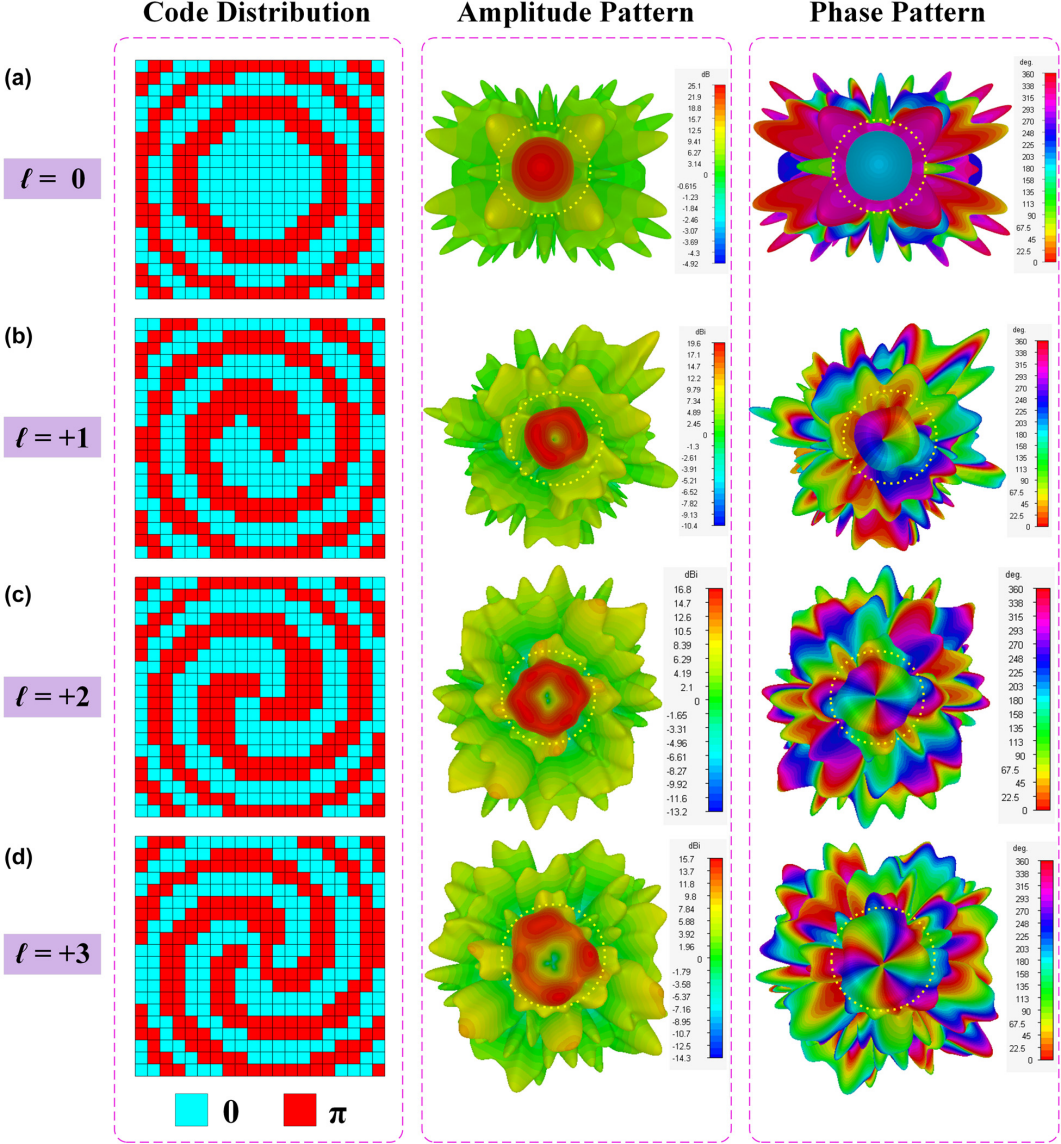
Code distributions and simulated radiation patterns for different OAM modes. (a) OAM mode *l* = 0. (b) OAM mode *l* = +1. (c) OAM mode *l* = +2. (d) OAM mode *l* = +3.

To make a primary verification for the proposed mmWave programmable metasurface, the generations of four converged vortex beams, including OAM modes *l* = 0, *l* = +1, *l* = +2, and *l* = +3, are numerically studied, as shown in Figure 5. The optimal quantized code distributions for the four modes are presented in the first column of Figure 5, which are performed according to Eq. (2) and clearly exhibit the vortex configurations for all nonzero OAM modes. The simulated far-field radiation amplitude patterns for the four OAM beams are provided in the second column of Figure 5, where conical-shaped patterns of high-intensity radiation are clearly presented for nonzero OAM modes, while a high-gain directional pencil beam is stimulated for the 0th mode. The simulated far-field phase patterns for the four modes are revealed in the third column of Figure 5. The typical vortex-shaped phase fronts along with on-axis phase singularities are clearly demonstrated for nonzero OAM modes. It is worth noting that, the radiation characters of the three negative-mode OAM beams, including modes *l* = −3, *l* = −2, and *l* = −1, could also be derived, whose phase profiles are directly opposite to those OAM beams with positive mode numbers, based on the conventional mirror image principle.

## 5 Experimental verification

**Figure 6:**
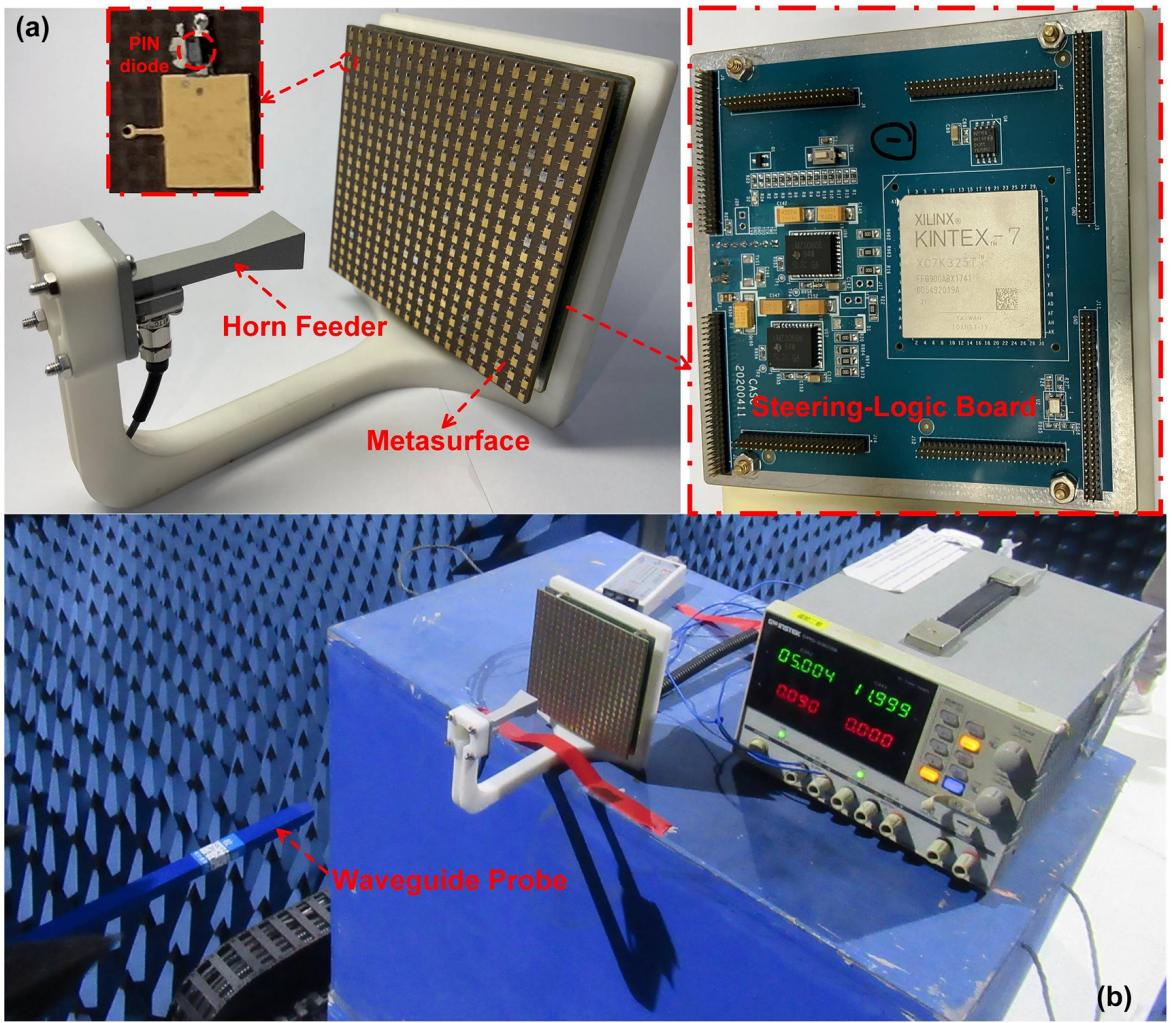
Fabricated prototype of the mmWave programmable metasurface. (a) Metasurface along with the horn feeder as well as the steering-logic board. (b) Experimental setup with a waveguide probe in near field chamber.

To further inspect and validate the actual functions of the proposed programmable metasurface for stimulating dynamical multi-mode mmWave OAM beams, the metasurface prototype with 20 × 20 units was manufactured. A pyramid horn feeder is introduced to illuminate the overall metasurface aperture, and a steering-logic board is integrated at the back of the metasurface. The metasurface along with horn feeder as well as the steering-logic board are all mounted onto a designed polyethylene bracket, as shown in Figure 6. The metasurface is connected and fixed with the steering-logic board through eight pairs of 1.27 mm SMT header connectors. In order to enhance the practicability of the proposed metasurface, the steering-logic board is carefully constructed with the same dimensions of the metasurface through miniaturized designs, thus a compact size can be obtained with the excellent expansibility for the larger metasurface array.

In order to obtain the dynamic OAM operation, the state of each PIN diode is modulated with the steering-logic board through the bias layer network. The whole steering-logic board is powered by an external voltage-stabilized source with a 5 V voltage input supply. The FPGA, Xilinx Kintex-7, is adopted as the host processing system in order to regulate the control signal in parallel for all 400 PIN diodes of the metasurface within the assigned clock signals, according to the optimal quantized code distributions. For the *π*-state, a designed bias current 10 mA along with a positive DC-biasing voltage +1.3 V are supplied for the PIN diode through the steering-logic board; for the 0-state, a negative DC-biasing voltage with neglectable current are provided. Since typically half of the 400 PIN diodes are operating at the *π*-state during the dynamic OAM modulation, the average power consumption of the steering-logic board can thus be estimated to be only 2.6 W.

The measurements of the proposed mmWave programmable metasurface are carried out by using a 3D platform in the near-field anechoic chamber as shown in Figure 6(b), and the performances of the generated OAM beams are calibrated accurately by employing an open-ended waveguide probe to acquire the near-field data in both amplitude and phase. In the measurement, the open-ended waveguide probe is placed behind the horn feeder with a distance of 270 mm away from the programmable metasurface, and both the open-ended probe and the horn feeder are connected to the two ports of the PNA network analyzer (Keysight N5222A). The near-field scanning plane is comprised of an overall range of 202.5 mm × 202.5 mm, and discretized into 45 × 45 sampling regions, with a scanning resolution less than half wavelength of 4.5 mm in both *x* and *y* directions. In order to verify the practical applicability of the proposed metasurface, the measured far-field radiation full-hemisphere amplitude and phase patterns are further calculated from the planar near-field measurements by using the Fourier transform integral [46].

**Figure 7:**
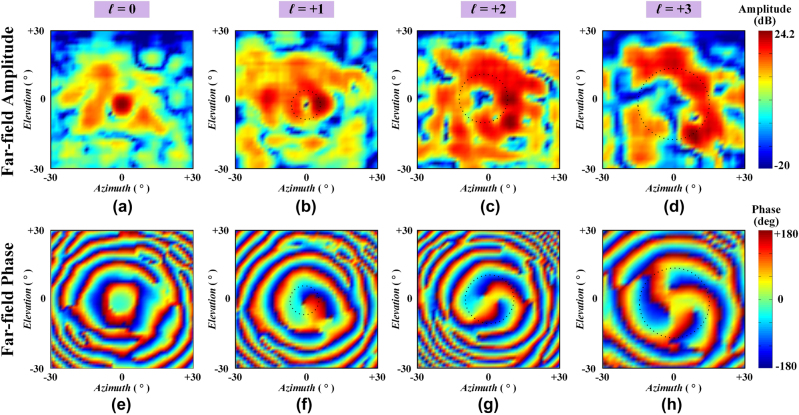
Far-field experimental results of the fabricated metasurface for different OAM modes. Amplitude: (a) *l* = 0, (b) *l* = +1, (c) *l* = +2, (d) *l* = +3. Phase: (e) *l* = 0, (f) *l* = +1, (g) *l* = +2, (h) *l* = +3.

Figure 7 presents the measured far-field amplitude and phase distributions for the four modes *l* = 0, *l* = +1, *l* = +2, and *l* = +3. As observed, a measured gain over 24.2 dB is stimulated for the zero-mode pencil beam, and the aperture efficiency is in excess of 20.9%. The characteristic vortex phase fronts as well as the on-axis phase singularities are exhibited for the nonzero OAM modes, indicating the effectiveness of the proposed programmable metasurface for dynamic mmWave OAM beam conversion, and the spatial area of the non-radiation region for the vortex beam is also growing as the OAM mode number increases. There is some aliasing shown in the far-field measured results when comparing with the simulated ones, which is primarily due to the blockage of the reflective waves caused by the polyethylene bracket. Such a problem is more severe for the mmWaves with shorter wavelengths. Another minor factor may attribute to the limited binary phase resolutions of the proposed 1 bit reconfigurable unit of the metasurface, where continuous phase variations are typically required. The total efficiency can be reduced, since the quantization loss for 1 bit metasurface is approximately 3 dB when comparing with that of continuous phase variations [47]. Nevertheless, our previous work of transmissive programmable metasurface [39] has verified that, despite a slight effect of the mode purity, the feasibility and validity of generating multi-mode OAM beams are undoubted even in a 1 bit programmable metasurface. To furtherly evaluate the OAM mode purity, the OAM mode spectra of the generated OAM beams have been calculated, and the Fourier relationship is employed according to the following equation [48],

(3) $$A_{l}=\frac{1}{2\pi }\int_{0}^{2\pi }\psi \left(\varphi \right)\text{d}\varphi {\text{e}}^{-jl\varphi }$$

(4) $$\psi \left(\varphi \right)=\sum_{l}A_{l}{\text{e}}^{jl\varphi }\text{.}$$

**Figure 8:**
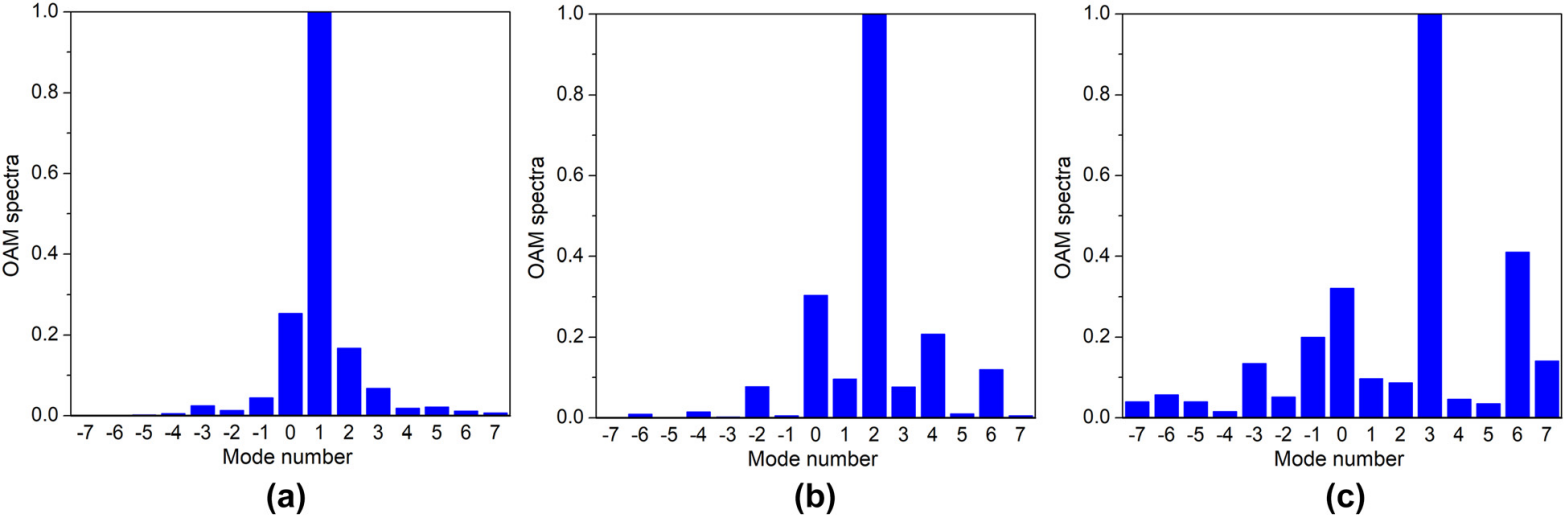
OAM spectra for different modes: (a) *l* = +1, (b) *l* = +2, (c) *l* = +3.

The OAM mode spectra are revealed as shown in Figure 8, and the desired OAM modes are in a relatively dominant position when comparing with the parasitic ones for all three OAM modes.

## 6 Conclusions

In summary, a reflective programmable metasurface constructed from reconfigurable units with 1 bit phase modulation has been presented for stimulating multi-mode mmWave vortex beams dynamically. Through configuration integration of a PIN diode within the radiation patch for modulating the unit resonance property, both low reflection losses and stabilized inverse phase states were obtained for the binary unit coding states within the operation mmWave band. Based on the proposed reconfigurable units, a dynamic programmable metasurface operating at 28–30 GHz with 20 × 20 units was designed and fabricated. The generation of four-mode vortex beams, including OAM modes *l* = 0, *l* = +1, *l* = +2, and *l* = +3, is dynamically stimulated via the steering-logic board, which demonstrates the effectiveness of our proposed design. The proposed mmWave OAM metasurface could be useful for the hybrid communications of both mmWave and multi-mode OAM technologies in the future.
